# Theoretical Research on Diffusion Radius of Cement-Based Materials Considering the Pore Characteristics of Porous Media

**DOI:** 10.3390/ma15217763

**Published:** 2022-11-03

**Authors:** Bao Xie, Hua Cheng, Xuesong Wang, Zhishu Yao, Chuanxin Rong, Ruihe Zhou, Liangliang Zhang, Longhui Guo, Hong Yu, Wei Xiong, Xusong Xiang

**Affiliations:** 1 School of Civil Engineering and Architecture, Anhui University of Science and Technology, Huainan 232001, China; 2 School of Resources and Environmental Engineering, Anhui University, Hefei 230601, China; 3 Anhui Province Key Laboratory of Building Structure and Underground Engineering, Anhui Jianzhu University, Hefei 230601, China; 4 School of Civil Engineering and Architecture, Anhui University of Technology, Ma’anshan 243032, China

**Keywords:** penetration grouting, tortuosity effect, time-varying viscosity, spherical diffusion

## Abstract

In engineering, loose sandy (gravelly) strata are often filled with cement-based grout to form a mixed material with a certain strength and impermeability, so as to improve the mechanical properties of sandy (gravelly) strata. The tortuosity effect of sandy (gravelly) strata and the time-varying viscosity of slurry play a key role in penetration grouting projects. In order to better understand the influence of the above factors on the penetration and diffusion mechanism of power-law slurry, based on the capillary laminar flow model, this research obtained the seepage motion equation of power-law slurry, the time-varying constitutive equations of tortuosity and power-law fluid viscosity were introduced, and the spherical diffusion equation of penetration grouting considering both the tortuosity of porous media and time-varying slurry viscosity was established, which had already been verified by existing experiments. In addition, the time-varying factors of grouting pressure, the physical parameters of the injected soil layer, and slurry viscosity on penetration grouting diffusion law and the influencing factors were analyzed. The results show that considering the tortuosity of sandy (gravelly) strata and the time-varying of slurry viscosity at the same time, the error is smaller than the existing theoretical error, only 13~19%. The diffusion range of penetration grouting in the sandy (gravelly) strata is controlled by the tortuosity of sandy (gravelly) strata, the water–cement ratio of slurry, and grouting pressure. The tortuosity of sandy (gravelly) strata is inversely proportional to the diffusion radius of the slurry, and the water–cement ratio of slurry and grouting pressure are positively correlated with the diffusion radius. In sandy (gravelly) strata with a smaller particle size, the tortuosity effect of porous media dominates the slurry pressure attenuation. When the particle size is larger, the primary controlling factor of slurry pressure attenuation is the tortuosity effect of porous media in the initial stage and the time-varying viscosity of slurry in the later stage. The research results are of great significance to guide the penetration grouting of sandy (gravelly) strata.

## 1. Introduction

Grouting technology is one of the important construction measures to block groundwater and reinforce weak stratum in urban underground engineering. Because the loose aquifer is mainly sandy (gravelly) strata, it is mostly the target layer for grouting, water sealing, and for the reinforcement of urban subway tunnels and deep excavation. Theoretical research is characterized by high efficiency, clear parameter meaning, and low research cost. However, because of the complexity of this kind of soil layer, the needs of engineering design and construction are difficult to satisfy through theoretical research, so it is urgent to carry out systematic and in-depth theoretical research [1].

Domestic and foreign scholars have made a series of research achievements in the properties of slurry and its diffusion law in sandy (gravelly) strata. Wu et al. [2], considering the tortuosity effect of porous media and combining the Hagen–Poiseuille equation, deduced a resistance model to describe the fluid flow in porous media, and pointed out that the flow resistance in porous media was mainly caused by the viscous energy loss of slurry at a low Reynolds number. Yun et al. [3] studied the tortuosity of streamline in porous media composed of particles of various shapes in two-dimensional and three-dimensional cases using geometric methods and explored the flow mechanism of various fluids in porous media. After that, Yun [4], and Wang et al. [5] studied the diffusion law of Bingham fluid in porous media based on the tortuous effect of porous media. Wang et al. [6], taking Bingham slurry as the carrier, derived the analytical solution of slurry pressure attenuation and diffusion radius considering the tortuosity of the injected loose layer, and verified the rationality of the theory through field grouting. Xiao et al. [7] and Han et al. [8] deduced the slurry diffusion model considering the pore tortuosity effect based on the pore tortuosity effect combined with the power-law fluid constitutive equation, and analyzed the influence law of the tortuosity effect, slurry viscosity, rheological index, and grouting amount per unit time on the attenuation of slurry pressure in the direction of the diffusion radius. The results show that the fractal characteristics of porous media have a significant influence on the pressure attenuation and diffusion radius of slurry. Ye et al. [9], Floar et al. [10], and Yang et al. [11,12] studied the column and column-hemisphere diffusion of power-law slurry in sandy (gravelly) strata based on penetration theory, but did not consider the time-varying viscosity of the slurry. Lv et al. [13], Zhang et al. [14], Cheng et al. [15], Liu et al. [16], Li et al. [17], Wang et al. [18], and Liu et al. [19], combined with theoretical analysis and experimental verification, explored the crack diffusion law of a single slab based on the time-varying viscosity of the slurry, and discussed the influence of the time-varying viscosity of slurry on fracture grouting. Guo et al. [20] used COMSOL numerical simulation software to verify the theoretical results of penetration grouting in sandy (gravelly) strata and analyzed the influence of the time-varying viscosity of slurry on the penetration diffusion of slurry in sandy (gravelly) strata. F. Bouchelachem [21,22] studied the filtration mechanism based on the particle adsorption probability model and found that the closer the injected area is to the grouting port, the more slurry particles are deposited.

To sum up, the existing research mainly focuses on the penetration diffusion of Newton and Bingham slurry in sandy (gravelly) strata, but there are few related studies on the penetration diffusion law in sandy (gravelly) strata considering the tortuosity effect and the time-varying viscosity of grout. The existing theory of penetration grouting in sandy (gravelly) strata seriously lags behind the engineering practice.

Therefore, the time-varying constitutive equation of tortuosity and power-law fluid viscosity was introduced, and the spherical diffusion equation of penetration grouting considering the tortuosity of porous media and the time-varying viscosity of the slurry was established. The time-space distribution equation of slurry pressure in the diffusion area and the theoretical calculation formula of diffusion radius were derived, and their rationality was verified according to the existing experimental results. In addition, the influence of strata tortuosity, slurry water–cement ratio, time-varying viscosity, grouting pressure, and other factors on the strata penetration grouting diffusion law was analyzed.

## 2. The Tortuosity Effect of Porous Media Considering the Influence of Slurry Particles on Porosity

### 2.1. Tortuosity Effect of Porous Media

Generally, tortuosity is defined as the ratio of the actual length of the fluid path to the straight line length or sample thickness along the macro pressure gradient direction [23,24], which is expressed by the following formula:

(1) $$\Gamma =\frac{L_{t}}{L_{0}}$$

where $L_{t}$ is the actual length of the fluid path, that is, $L_{0}$ is the straight line length along the macro pressure gradient direction or the thickness of the sample. The particles in porous media are simplified into sphericity. Considering that Comiti [25] has passed the particle bed fluid test, the empirical formula between the tortuosity and porosity of fluid passing through porous media composed of spherical particles is obtained as follows:

(2) $$\Gamma \left(\phi \right)=1+0.41\ln\left(1/\phi \right)$$

**Figure 1:**
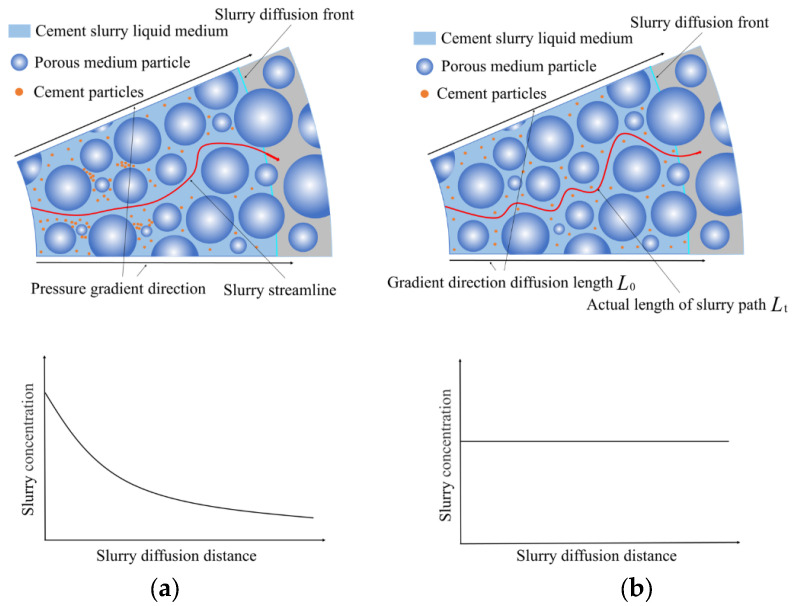
Comparison of different tortuous models: (**a**) uneven filtration tortuous model; [1] (**b**) simplified tortuous model.

In the process of grouting, the filtration effect of porous media on slurry particles is spatial non-uniformity [26,27,28,29]. As shown in Figure 1a, this characteristic will increase the penetration resistance of slurry in porous media, leading to the acceleration of pressure attenuation of slurry in porous media. To introduce the filtration effect into this study, a simplified model is proposed, as shown in Figure 1b. The actual length of the seepage path of the slurry in the grouting process is $L_{t}$, and the effective diffusion distance is $L_{0}$. It is assumed that the cement particles are uniformly suspended in the injected media, and the subsequent grouting will pass through the porous media particles and suspended cement particles to reach the non-grouting area. The advantage of this model is that the resistance caused by the filtration effect in porous media is included in the increment of tortuosity through the homogenization hypothesis, which is convenient to be introduced into this study. The actual porosity of the grout passing through the grouted area is smaller than that of the original porous media, so the tortuosity of the grouting process is larger than that of the original porous media, which replaces the blocking effect of the grout particle filtration effect on the subsequent grout injection.

The volume of cement particles with conservation of mass in the grouting area is as follows:

(3) $$V_{c}=\frac{m_{c}}{\rho_{c}}=\frac{\rho_{s}\cdot Q}{\rho_{c}\left(W/C+1\right)}=\frac{\rho_{s}}{\rho_{c}\left(W/C+1\right)}\cdot \frac{4}{3}\pi l^{3}\cdot \phi$$

where $V_{c}$ is the volume of cement particles in the grouting area; $m_{c}$ is the mass of cement particles; $\rho_{c}$ is the density of cement particles; $\rho_{s}$ is the density of slurry, the specific gravity of cement slurry with a water–cement ratio of 0.5, 0.6, and 0.7 is 1.835, 1.744, and 1.671, respectively; Q is the grout amount; W/C is the water–cement ratio of slurry; l is the radius of spherical diffusion area of slurry in porous media; and ϕ is the porosity of the porous media.

### 2.2. Effect of Slurry Particles on Porosity of Porous Media

Without considering the influence of the slurry particle injection on the porosity of the porous media, the porosity of the porous media can be expressed by Equation (4):

(4) $$\phi =1-\frac{V_{p}}{V_{p}+V_{c}+V_{w}}$$

**Figure 2:**
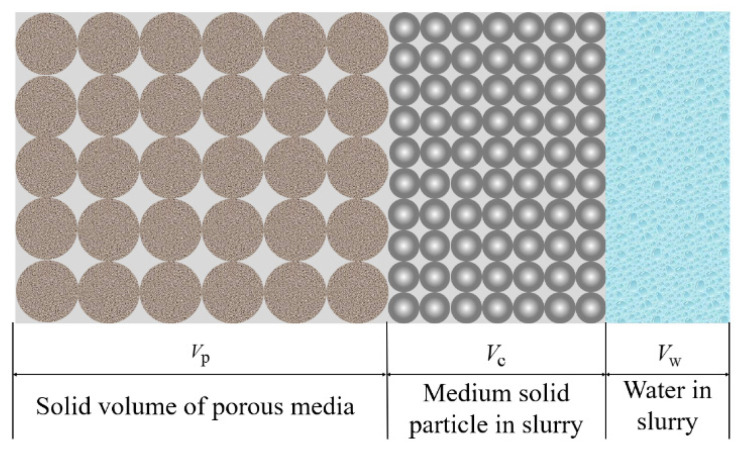
Volume distribution of each component in the grouting area.

Considering the influence of slurry particles on porosity, through the homogenization hypothesis (as shown in Figure 2), the actual average porosity ϕ′ in the injected porous media can be obtained as follows:

(5) $$\phi \prime =1-\frac{V_{p}+V_{c}}{V_{p}+V_{c}+V_{w}}=\phi -\frac{V_{c}}{\frac{4}{3}\pi l^{3}}=\left[1-\frac{\rho_{s}}{\rho_{c}\left(W/C+1\right)}\right]\cdot \phi$$

It can be obtained from the Equation (5) that cement particles in slurry become a part of porous media due to the adsorption and accumulation of cement particles in slurry [30,31,32]. Therefore, when considering the tortuosity of porous media, the actual porosity ϕ′ should be adopted. The actual average tortuosity $\Gamma \left(\phi \prime \right)$ corresponding to it is as follows:

(6) $$\Gamma \left(\phi \prime \right)=1+0.41\ln\left(1/\phi \prime \right)$$

The pressure attenuation of laminar slurry is mainly caused by the viscous resistance between the slurry and porous media. The greater the tortuosity of the porous media, the greater the contact surface between the slurry and porous media [2], the greater the viscous resistance, and the more obvious the attenuation of slurry pressure. Considering the influence of cement particles in the above grouting slurry on the porosity of porous media, it was found that as the slurry was injected into porous media, the pores of the porous media were filled by cement particles in the slurry, and the porosity decreased, which increased the tortuosity of the porous media in the grouting process and intensified the pressure attenuation of the subsequent slurry in the diffusion process.

## 3. Diffusion Model of Soil Penetration Grouting Based on Tortuosity Effect and Time-Varying Viscosity of the Slurry

### 3.1. Basic Hypothesis

(1) The soil layer is regarded as a porous media composed of spherical particles with different particle sizes, and the cement particles in the slurry are sphericity with the same particle size;
(2) When slurry flows in porous media, the uneven adsorption and deposition of solid particles is simplified as slurry uniformly dispersed in the injected area;
(3) Slurry is regarded as an incompressible fluid and still maintains a power-law flow pattern with time-varying viscosity during grouting [20];
(4) The flow of power-law slurry in porous media is regarded as laminar flow, and the energy loss of slurry at the capillary bend in the porous media is ignored [3,33].

### 3.2. Constitutive Equation of Power Law Slurry Considering Time-Varying Viscosity

The rheological equation of power-law slurry without considering the time-varying viscosity is as follows [34,35]:

(7) $$\tau =C\cdot \gamma^{n}$$

where τ is shear stress; C is the viscosity coefficient; n is the rheological index; and γ is the shear rate, γ=−dv/dr.

According to the research results of Ruan [34], when considering the time-varying viscosity of the slurry, the viscosity coefficient of the power-law slurry can be expressed as an exponential function with time as the independent variable, which reflects the trend that the viscosity of the power-law slurry gradually increases with time. However, the change of rheological index with time can be neglected, which can be regarded as the time-invariant in engineering practice. The viscosity coefficient and rheological index of power-law slurry can be summarized as follows:

(8) $$C=C\left(t\right)=C_{0}e^{\lambda t}$$

(9) $$n=n\left(t\right)=n_{0}$$

where $C_{0}$ is the initial viscosity coefficient and λ is the coefficient representing the time-varying viscosity.

The rheological constitutive equation of the power-law slurry considering time-varying viscosity can be obtained by combining Equations (7)–(9) as follows:

(10) $$\tau \left(t\right)=C_{0}e^{\lambda t}\cdot \gamma^{n_{0}}$$

### 3.3. Laminar Flow of the Power-Law Slurry in the Capillary

**Figure 3:**
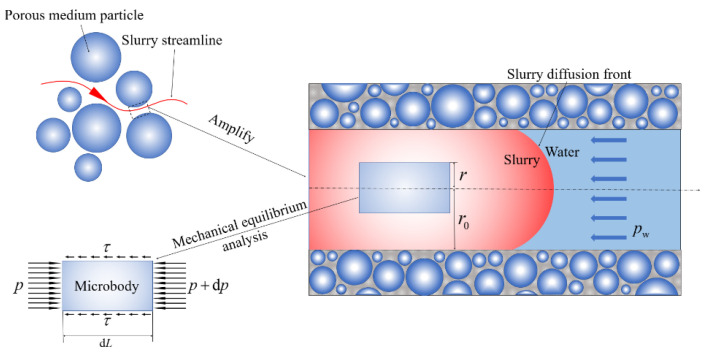
Unit-volume equilibrium analysis of the power-law fluid in the capillary.

The flow of slurry in the porous media is regarded as the laminar flow of the capillary. Take a circular tube with radius $r_{0}$, and select the stress state of a microfluidic column with radius $r\left(r<r_{0}\right)$ and length dL, as shown in Figure 3. Without considering the gravity of the slurry, the equation of the force balance in the horizontal direction of the micro-fluid column is as follows [18,36]:

(11) $$\pi r^{2}dp+2\pi r\tau dL=0$$

The pressure at both ends of the microfluidic column is p+dp and p, respectively, and the shear stress on its surface is τ, and the direction is opposite to the direction of the movement rate of the microfluidic column.

According to Equation (11), the shear stress on the surface of the micro-fluid column is as follows:

(12) $$\tau =-\frac{r}{2}\frac{dp}{dL}$$

Substituting Equation (12) into the rheological constitutive equation of the power-law slurry considering the time-varying viscosity can be obtained as follows:

(13) $$\gamma =-\frac{dv}{dr}={\left[\frac{\tau }{C_{0}e^{\lambda t}}\right]}^{\frac{1}{n_{0}}}={\left[-\frac{1}{2C_{0}e^{\lambda t}}\cdot \frac{dp}{dL}\right]}^{\frac{1}{n_{0}}}r^{\frac{1}{n_{0}}}$$

Equation (13) can be obtained by integrating the method of separating variables:

(14) $$v=-{\left[-\frac{1}{2C_{0}e^{\lambda t}}\cdot \frac{dp}{dL}\right]}^{\frac{1}{n_{0}}}\frac{n_{0}}{1+n_{0}}r^{\frac{1+n_{0}}{n_{0}}}+C_{1}$$

Consider the boundary condition: when the slurry velocity at the capillary wall is 0, that is, when $r=r_{0}$, v=0. According to this, the integral constant $C_{1}$ is obtained and substituted into Equation (14) to obtain the following:

(15) $$v=-{\left[-\frac{1}{2C_{0}e^{\lambda t}}\cdot \frac{dp}{dL}\right]}^{\frac{1}{n_{0}}}\frac{n_{0}}{1+n_{0}}\left(r_{0}{}^{\frac{1+n_{0}}{n_{0}}}-r^{\frac{1+n_{0}}{n_{0}}}\right)$$

The equation is the temporal and spatial distribution of the slurry velocity in the capillary, and the flow rate per unit time through the capillary with radius $r_{0}$ can be expressed as follows:

(16) $$q=\int_{0}^{r_{0}}2\pi rvdr$$

Substituting Equation (15) into Equation (16), the flow rate q per unit time through the capillary can be obtained as follows:

(17) $$q=\frac{\pi n_{0}}{1+3n_{0}}{\left[-\frac{1}{2C_{0}e^{\lambda t}}\cdot \frac{dp}{dL}\right]}^{\frac{1}{n_{0}}}r_{0}{}^{\frac{1+3n_{0}}{n_{0}}}$$

It can be obtained that the average flow rate $\bar{v}$ on the capillary section with the cross-sectional area *S* is:

(18) $$\bar{v}=\frac{q}{S}=\frac{q}{\pi r_{0}{}^{2}}=\frac{n_{0}}{1+3n_{0}}{\left[-\frac{1}{2C_{0}e^{\lambda t}}\cdot \frac{dp}{dL}\right]}^{\frac{1}{n_{0}}}r_{0}{}^{\frac{1+n_{0}}{n_{0}}}$$

In the above equation, *L* is the length of the capillary, while the streamline of slurry in porous media is a curve, and the effective diffusion length of the slurry in porous media is *l*. According to the definition of tortuosity, there is the following relationship:

(19) $$L=\Gamma \left(\phi \prime \right)\cdot l$$

Differentiate both sides of the equation at the same time:

(20) $$dL=\Gamma \left(\phi \prime \right)dl$$

The effective seepage velocity of the power-law slurry in the porous media with the actual porosity of ϕ′ in the grouting process can be expressed as follows:

(21) $$V=\phi \prime \bar{v}=\frac{\phi \prime n_{0}}{1+3n_{0}}{\left[-\frac{1}{2C_{0}e^{\lambda t}}\cdot \frac{1}{\Gamma \left(\phi \prime \right)}\cdot \frac{dp}{dl}\right]}^{\frac{1}{n_{0}}}r_{0}{}^{\frac{1+n_{0}}{n_{0}}}$$

Introducing effective viscosity $\mu_{e}$ and effective permeability $K_{e}$ of power-law fluid [20]:

(22) $$\mu_{e}=C_{0}{\left(\frac{1+3n_{0}}{\phi \prime r_{0}n_{0}}\right)}^{n_{0}-1}$$

(23) $$K_{e}=\frac{\phi \prime r_{0}{}^{2}}{2}\left(\frac{n_{0}}{1+3n_{0}}\right)$$

Considering the time-varying viscosity of the power-law slurry, the corresponding effective viscosity also has time-varying properties, and the effective viscosity at this time is expressed as follows:

(24) $$\mu_{e}\left(t\right)=C_{0}e^{\lambda t}{\left(\frac{1+3n_{0}}{\phi \prime r_{0}n_{0}}\right)}^{n_{0}-1}$$

Substituting Equations (22) and (23) into Equation (24) for simplification, the effective seepage velocity *V* of the power-law slurry in the porous media is as follows:

(25) $$V={\left[\frac{K_{e}}{\mu_{e}\left(t\right)}\right]}^{\frac{1}{n_{0}}}{\left(-\frac{1}{\Gamma \left(\phi \prime \right)}\cdot \frac{dp}{dl}\right)}^{\frac{1}{n_{0}}}$$

### 3.4. Diffusion Equation of Spherical Penetration Grouting with Power-Law Slurry

**Figure 4:**
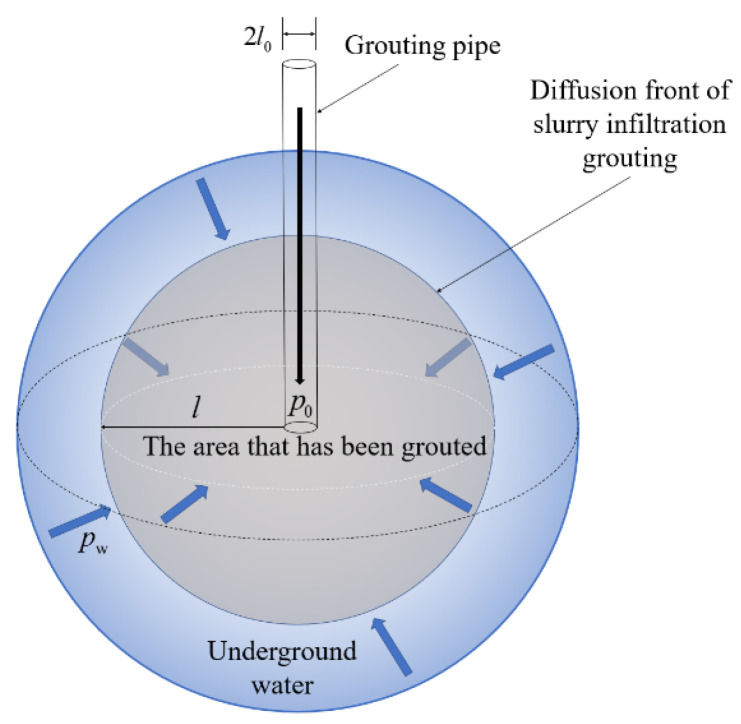
Theoretical model of the slurry spherical diffusion.

The theoretical model of spherical penetration and diffusion of cement slurry in porous media is shown in Figure 4, in which $2l_{0}$ is the grouting pipe diameter, l is the slurry diffusion radius, $p_{0}$ is grouting pressure, and $p_{w}$ is the groundwater pressure.

The grouting quantity *Q* of slurry in spherical diffusion in porous media should meet the following requirements:

(26) Q=VAt

where *A* is the area of slurry diffusion front when the grouting time is *t*.

The vertical Equations (25) and (26) can be obtained as follows:

(27) $$\frac{Q}{4\pi l^{2}t}={\left[\frac{K_{e}}{\mu_{e}\left(t\right)}\right]}^{\frac{1}{n_{0}}}{\left(-\frac{1}{\Gamma \left(\phi \prime \right)}\cdot \frac{dp}{dl}\right)}^{\frac{1}{n_{0}}}$$

By integrating Equation (27) with separated variables:

(28) $$p=-\Gamma \left(\phi \prime \right){\left(\frac{Q}{4\pi t}\right)}^{n_{0}}\left[\frac{\mu_{e}\left(t\right)}{K_{e}}\right]\left(\frac{l^{1-2n_{0}}}{1-2n_{0}}\right)+C_{2}$$

Considering that the slurry pressure near the grouting port is the grouting pressure, that is, when $l=l_{0}$, $p=p_{0}$.

When the slurry diffusion front and the grouting port are $l_{1}$, the slurry pressure is $p_{1}$, that is, when $l=l_{1}$, $p=p_{1}$.

By substituting the above boundary conditions into Equation (28), the grout content in the grouting sphere with radius $l_{1}$ is considered as the total seepage flow of the grout:

(29) $$Q=\frac{4}{3}\pi l_{1}{}^{3}\phi \prime$$

The slurry pressure at $l_{1}$ from the grouting port and the pressure difference at the grouting port are as follows:

(30) $$\Delta p=p_{0}-p_{1}=\Gamma \left(\phi \prime \right){\left(\frac{\phi \prime }{3t}\right)}^{n_{0}}\left[\frac{\mu_{e}\left(t\right)}{K_{e}}\right]\left(\frac{1}{1-2n_{0}}\right)\left(l_{1}{}^{1-2n_{0}}-l_{0}{}^{1-2n_{0}}\right)l_{1}{}^{3n_{0}}$$

In the above equation, $l_{1}$ is replaced by l to obtain the slurry pressure at any distance l from the grouting port at t time, as follows:

(31) $$p=p_{0}-\Delta p=p_{0}-\Gamma \left(\phi \prime \right){\left(\frac{\phi \prime }{3t}\right)}^{n_{0}}\left[\frac{\mu_{e}\left(t\right)}{K_{e}}\right]\left(\frac{1}{1-2n_{0}}\right)\left(l^{1-2n_{0}}-l_{0}{}^{1-2n_{0}}\right)l^{3n_{0}}$$

Substituting Equations (23) and (24) into Equation (31) gives the following:

(32) $$p\left(t,l\right)=p_{0}-\frac{2\Gamma \left(\phi \prime \right)}{r_{0}\left(1-2n_{0}\right)}{\left(\frac{1+3n_{0}}{3r_{0}n_{0}}\right)}^{n_{0}}\frac{C_{0}e^{\lambda t}}{t^{n_{0}}}\left(l^{1-2n_{0}}-l_{0}{}^{1-2n_{0}}\right)l^{3n_{0}}$$

The above equation is the time-space variation equation of the slurry pressure in the spherical grouting area considering the time-varying viscosity of the power-law slurry.

Equation (32) can be used when the slurry pressure is the same as the water pressure, that is, when $p=p_{w}$, the implicit expression of the slurry diffusion radius at t time is as follows:

(33) $$l\left(t\right)={\left(\frac{3r_{0}n_{0}}{1+3n_{0}}\right)}^{\frac{1}{3}}{\left[\frac{r_{0}\left(1-2n_{0}\right)}{2\Gamma \left(\phi \prime \right)}\frac{t^{n_{0}}}{C_{0}e^{\lambda t}}\cdot \left(p_{0}-p_{w}\right)\right]}^{\frac{1}{3n_{0}}}{\left[l{\left(t\right)}^{1-2n_{0}}-l_{0}{}^{1-2n_{0}}\right]}^{-\frac{1}{3n_{0}}}$$

When the diffusion radius of the slurry is close to the final area, $l\left(t\right)\gg l_{0}$, and because $n_{0}$ is less than 0.5 [37], Equation (32) can be simplified as follows:

(34) $$p=p_{0}-\frac{2\Gamma \left(\phi \prime \right)}{r_{0}\left(1-2n_{0}\right)}{\left(\frac{1+3n_{0}}{3r_{0}n_{0}}\right)}^{n_{0}}\frac{C_{0}e^{\lambda t}}{t^{n_{0}}}l^{1+n_{0}}$$

Similarly, the diffusion radius of the slurry can be expressed as follows:

(35) $$l\left(t\right)=\frac{3r_{0}n_{0}}{1+3n_{0}}{\left[\frac{\left(1-2n_{0}\right)\left(1+3n_{0}\right)}{6n_{0}\Gamma \left(\phi \prime \right)}\cdot \frac{t^{n_{0}}}{C_{0}e^{\lambda t}}\cdot \left(p_{0}-p_{w}\right)\right]}^{\frac{1}{1+n_{0}}}$$

Equations (34) and (35) describe the law of slurry pressure attenuation of the power-law fluid in porous media when considering the slurry viscosity, and calculate the slurry diffusion radius corresponding to different times.

## 4. Verification Analysis

Table 1 and Table 2 show the relevant parameters of the penetration grouting in the literature [20], in which gravel soil is used as the injected media, and the radius of the grouting pipe is 7.5 mm. The gravel soil particles are classified as 1–3 mm, 3–5 mm, and 5–10 mm; the water–cement ratio is 0.7, 0.6, and 0.5; and the grouting pressure is 306.1 kPa, 459.2 kPa, and 612.2 kPa, respectively.

As can be seen from Table 2, the actual porosity in the grouting process is lower than the natural porosity of the porous media, and correspondingly, the tortuosity in the grouting process is 11.14%, 13.13%, and 15.78% higher than that of the porous media, respectively. It shows that considering the filtration effect of porous media on slurry through the homogenization hypothesis increases the resistance of slurry permeation and diffusion, which is consistent with the existing research results.

To verify the correctness of the diffusion of Equation (35) for the power-law slurry spherical infiltration grouting, the aforementioned theory, the existing theory, and the experimental measured value [20] are compared. The comparison between the theoretical values and measured values of the four theories under three working conditions is shown in Table 3 by substituting various parameters. L1, L2, L3, and L4 represent the theoretical values without considering tortuosity and viscosity time-varying, only considering the slurry viscosity time-varying, only considering tortuosity and considering tortuosity and time-varying viscosity, respectively.

**Figure 5:**
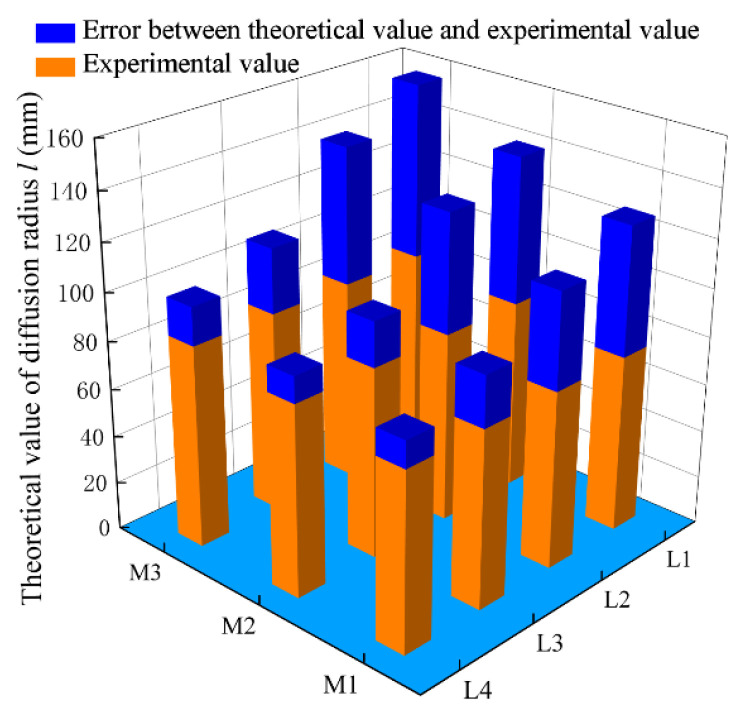
Different theoretical predicted values and their error analysis under three working conditions.

According to Table 3, the relative errors between different theoretical and measured values are analyzed, as shown in Figure 5.

Figure 5 shows that for the same working condition, with the improvement in the theory, the error between the theoretical value and the experimental value gradually decreased. In M1, when the time-varying viscosity of the slurry and the tortuosity of gravel soil media were not considered, the error was the largest, which was 53.29 mm, followed by the time-varying viscosity of the slurry and the tortuosity of the porous media, which were 39.86 mm and 20.95 mm, respectively. When both factors were considered, the error was only 14.74% of the measured value. For working conditions M2 and M3, the calculated values considering both factors were 13.57% and 18.61% higher, respectively, than the measured values. Therefore, it is reasonable to consider the tortuous effect of the porous media and the time-varying viscosity of the slurry.

## 5. Penetration and Diffusion Law of Grouting and Its Influencing Factors

Based on the above theoretical model, the time-varying factors of grouting pressure, the physical parameters of the injected soil layer, and slurry viscosity are studied to analyze the diffusion law and influencing factors of penetration grouting.

### 5.1. Slurry Pressure Attenuation Law

#### 5.1.1. Compared with Existing Theories

**Figure 6:**
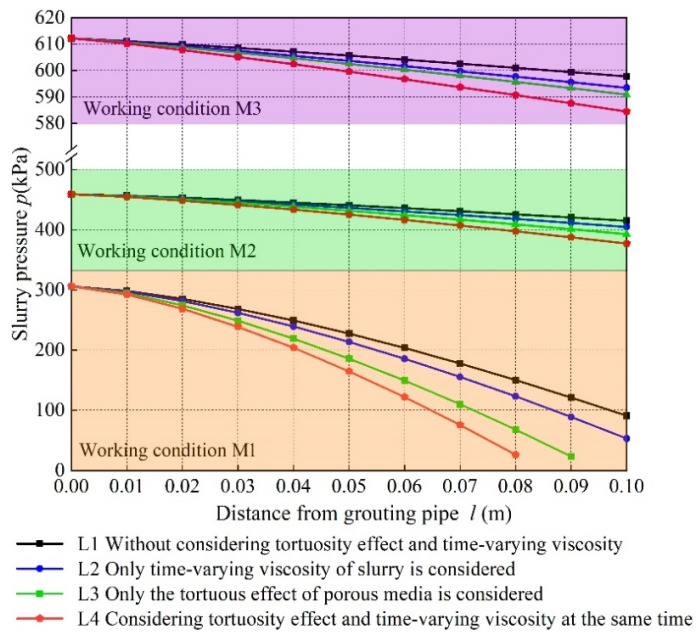
Attenuation law of the slurry pressure.

By substituting the parameters in Table 1 and Table 2 into Equation (34), the attenuation curve of the slurry pressure in the direction of diffusion radius is shown in Figure 6.

According to Figure 6, the slurry pressure decreases with the increase in the diffusion radius, and the pressure attenuation trend shows obvious nonlinear characteristics, and the slurry pressure attenuation rate is positively correlated with the grouting hole distance. When the time-varying viscosity (L1) of the slurry is not considered, the obstruction of slurry flow in the porous media is less than the real situation, and the slurry pressure attenuates slowly during diffusion. Considering the time-varying viscosity of the slurry, the viscosity of the power-law slurry increases exponentially with time. However, because of the small viscosity coefficient and short grouting time, the influence of the time-varying viscosity of the slurry on the pressure attenuation is smaller than that of the tortuosity of the porous media. This effect shows that the attenuation of slurry pressure predicted by theory L3 in Figure 4 is obviously larger than that predicted by theory L2. Under working condition M1, the pressure attenuation predicted by theory L2 is 17.59% faster than that predicted by theory L1, while the pressure attenuation predicted by theory L3 is 52.98% faster than that predicted by theory L1, which is 3.01 times that of only considering the time-varying factors of the slurry viscosity. For working conditions M2 and M3, this value is 2.15 times and 1.59 times, respectively. Therefore, for predicting the pressure attenuation in the process of injecting the power-law slurry into the porous media, the tortuosity effect of the porous media cannot be ignored. For working conditions M1, M2, and M3, due to the short grouting time and the tortuosity effect of the porous media, the attenuation of the slurry pressure in space is more obvious.

Under working condition M1, the injectability of the porous media is low because of its fine particles, low porosity, and high tortuosity. The grouting time of the three conditions is similar, and the pressure attenuation trend of M1 is more obvious than that of M2 and M3. As shown in Figure 5, the pressure attenuation curve and the abscissa quickly intersect. Under the M1 condition, the grouting pressure is constant, and the slurry quickly reaches the maximum diffusion radius. After that, even if the grouting time is prolonged, the diffusion radius of the slurry will not change significantly. Therefore, for porous media with fine particles and poor injectability, its slurry pressure attenuation is mainly determined by the tortuosity effect of the porous media. However, in working conditions M2 and M3, the attenuation trend of the slurry pressure tends to be gentle because of the larger particle size, larger porosity, smaller tortuosity, higher injectability, and lower resistance of slurry in the porous media.

#### 5.1.2. Analysis of Main Control Factors of Pressure Attenuation

**Figure 7:**
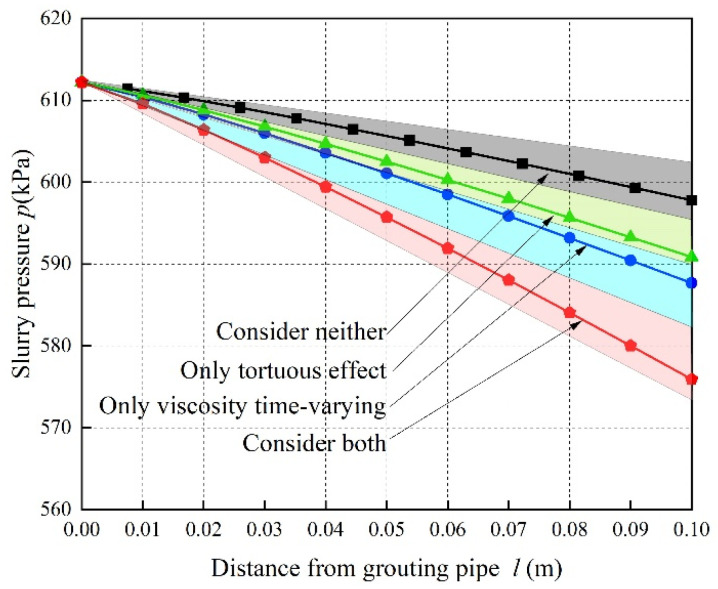
Attenuation law of slurry pressure.

The grouting time of M1, M2, and M3 is short, and the tortuosity effect of porous media dominates the slurry pressure attenuation. With the background of M3, the grouting time is increased from 240 s to 600 s, and the other parameters are unchanged. It is not difficult to obtain the pressure attenuation law curves described by the four theories, as shown in Figure 7.

From Figure 7, it can be seen that the pressure attenuation rate predicted by only considering the time-varying viscosity of the slurry is 70.09% faster than that predicted by not considering the time-varying viscosity of the slurry and the tortuosity of the porous media, while that predicted by only considering the tortuosity of the porous media is 48.48% faster than that predicted by not considering the time-varying viscosity of the slurry, which is only 69.17% of that for considering the time-varying viscosity of the slurry. Therefore, it is not difficult to see that for the long grouting time, the time-varying viscosity of the power-law grout dominates the spatial attenuation of the grouting pressure.

Because of the different influences of time-varying slurry viscosity and the tortuosity of porous media on the predicted pressure attenuation law of working condition M3, there is a certain intermediate moment that makes the sensitivity of the pressure attenuation of working condition M3 approximately that of the two influencing factors. Substituting the relevant parameters, it is found that the sensitivity of the slurry pressure attenuation of working condition M3 to the tortuosity of the porous media and time-varying slurry viscosity is similar when the grouting time is about 480 s.

Therefore, for working conditions M2 and M3, the main control factors of slurry pressure attenuation are different under the same working conditions and different grouting times: when the grouting time is short, the tortuous effect of the porous media is the main control factor of the slurry pressure attenuation, which is mainly reflected by the pressure attenuation predicted by theory L3 being more obvious than that predicted by theory L2. When the grouting time is long, the pressure attenuation of the grout is more sensitive to the time-varying viscosity of the grout, which is reflected by the pressure attenuation predicted by theory L2 being more significant than that predicted by theory L3.

To sum up, it is particularly important to consider the tortuosity of the porous media and the time-varying viscosity of the slurry. During grouting, the pressure attenuation of the slurry is affected by the joint action of the slurry and the porous media, and the cement particles in the slurry are evenly distributed in the porous media and become a part of the porous media, which increases the tortuosity of the porous media and hinders the subsequent slurry from entering the porous media. However, the tortuosity of the porous media increases the streamline length of the slurry in the direction of the diffusion radius, and the time-varying effect of the slurry viscosity is more obvious, which makes the slurry pressure attenuation more severe. Therefore, considering the above two factors is more in line with the attenuation law of the actual slurry pressure in space.

### 5.2. Variation Law of Diffusion Radius

#### 5.2.1. Influence of Tortuosity of Porous Media on Grouting Diffusion Radius

**Figure 8:**
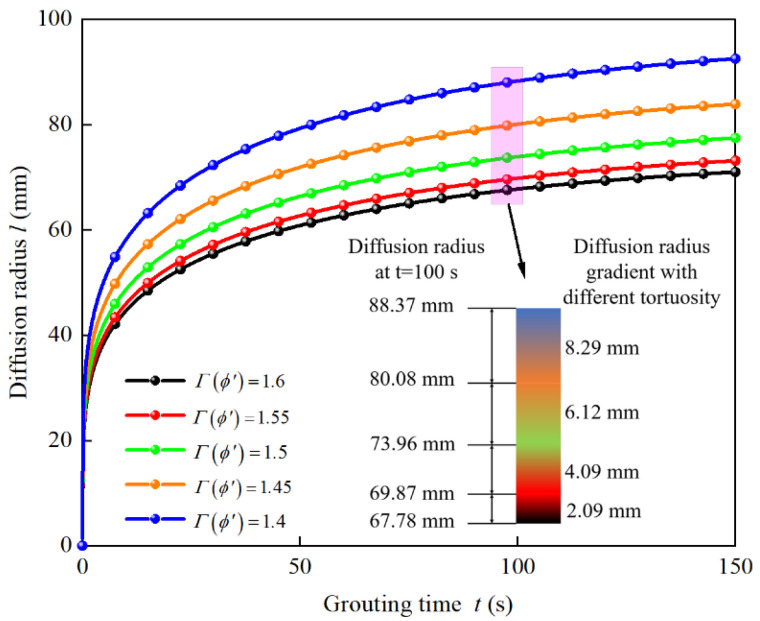
Influence of tortuosity on the slurry diffusion radius.

Take the water–cement ratio of the cement slurry as W/C = 0.6, the grouting pressure is 0.5 MPa and the tortuosity of the injected media in the grouting process is 1.4, 1.5, and 1.6, respectively. Add the parameters into Equation (35) to obtain the influence of the tortuosity of the injected media on the diffusion law of the penetration grouting, as shown in Figure 8.

It can be seen from Figure 8 that at the initial stage of grouting, the diffusion rate of the slurry is relatively fast, and gradually slows down with the grouting time. This is because with the increase in grouting time, the viscosity of the slurry increases, the resistance of the subsequent grouting slurry increases, and the diffusion rate of the slurry slows down. The greater the tortuosity of the grouting process, the smaller the diffusion radius of the slurry at the same time; the equal gradient increases the tortuosity; and the decreasing gradient of the diffusion radius at the same time shows a decreasing trend. This is because the greater the tortuosity, the lower the porosity of the injected soil layer, the more complex the pore structure, the greater the resistance of the gravel layer to the slurry, and the smaller the pore diameter, making it more difficult for the slurry to be injected into the porous media. Therefore, the narrowing gradient of the diffusion radius shows a decreasing trend.

#### 5.2.2. Influence of Water–Cement Ratio of Slurry on Diffusion Radius

**Figure 9:**
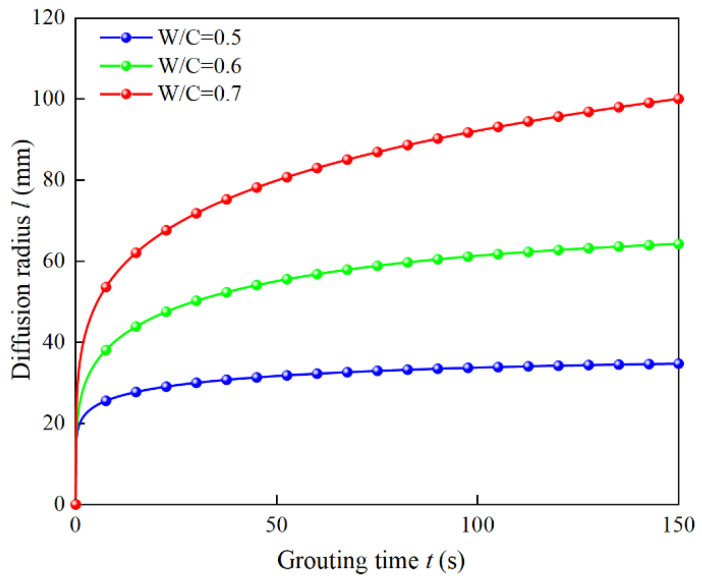
Influence of the water–cement ratio of the slurry on the diffusion radius.

The natural porosity of the injected soil layer is 0.45; the grouting pressure is 0.4 MPa; and the water–cement ratio of the cement slurry is 0.5, 0.6, and 0.7, respectively. From the above analysis, the water–cement ratio has two influences on grouting, one is to change the tortuosity of the soil layer, and the other is the different constitutive parameters of grout with different water–cement ratios. According to Equations (5) and (6), the porosity of the grouting process with a water–cement ratio of 0.5, 0.6, and 0.7 is 0.2748, 0.2937, and 0.3096, respectively, and the tortuosity of the grouting process is 1.5296, 1.5023, and 1.4807, respectively. Bringing the parameters into Equation (35), we can analyze the influence of the slurry water–cement ratio on the penetration and diffusion law, as shown in Figure 9.

It can be seen from Figure 9 that when the water–cement ratio is 0.5 and 0.6, the diffusion rate decays rapidly, and the diffusion radius tends to become stable quickly. When the water–cement ratio is 0.7, the diffusion rate decays slowly, and the diffusion radius of the grout still develops significantly in the later stage of grouting. Combined with the above theoretical analysis, the water–cement ratio has two influences on grouting. First, the injection of slurry particles changes the porosity of the gravel soil, resulting in the actual tortuosity of the grouting process; second, the constitutive parameters of the slurry with different water–cement ratios are different. For the slurry with a small water–cement ratio, its initial viscosity and time-varying parameters are large, so it is easy to reach the diffusion radius threshold. However, the slurry with a higher water–cement ratio is easier to inject into the injected soil layer. Therefore, the water–cement ratio of the slurry is closely related to the viscosity, and the corresponding constitutive parameters also have an important influence on the effect of penetration grouting.

#### 5.2.3. Influence of Grouting Pressure on Diffusion Radius

**Figure 10:**
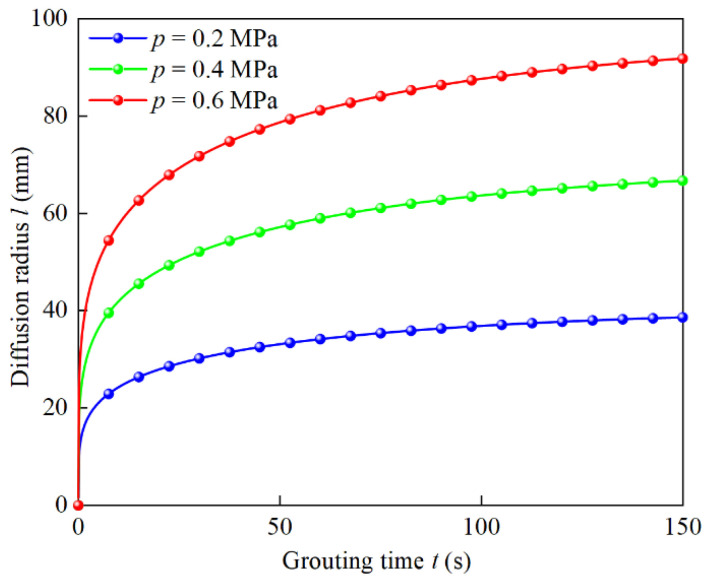
Influence of grouting pressure on diffusion radius.

Cement slurry with a water–cement ratio of 0.6 is injected into the soil layer with a natural porosity of 0.45. The porosity of the grouting process is 0.2937 from Equation (5), and the tortuosity of the grouting process is 1.5023 from Equation (6). The grouting pressures are 0.2 MPa, 0.4 MPa, and 0.6 MPa, respectively. Taking the parameters into Equation (35), the variation law of slurry diffusion radius with time under different grouting pressures is shown in Figure 10.

It can be seen from Figure 10 that when the grouting pressure is 0.2 MPa, the diffusion radius of 30% of the grouting time is 33.14 mm, which is 86.84% of the total diffusion radius. When the grouting pressure is 0.4 MPa and 0.6 MPa, the diffusion radius of 30% grouting time is 57.19 mm and 78.72 mm, reaching 86.36% and 85.71% of the total diffusion radius, respectively. Therefore, with the increase in grouting pressure, the attenuation trend of the slurry diffusion rate decreases slightly, and overall, the slurry diffusion rate decreases significantly in the early stage of grouting, resulting in a fixed diffusion range in the late stage of grouting, and the increasing grouting pressure significantly increases the diffusion radius of the slurry in the same grouting time. When the grouting time is 150 s, the diffusion radius is 38.62 mm, 66.69 mm, and 91.79 mm, respectively. When the grouting pressure is 0.4 MPa, the diffusion radius is 1.73 times for 0.2 MPa, and when the grouting pressure is 0.6 MPa, the diffusion radius is 2.38 times for 0.2 MPa. In practice, the grouting pressure can be reasonably increased according to the porosity of the injected soil layer, so that the slurry can quickly penetrate and diffuse in the soil layer to form an effective water-blocking curtain.

## 6. Conclusions

(1) By constructing the actual tortuosity model $\Gamma \left(\phi \prime \right)$ considering the uniform distribution of the slurry solid particles in sandy (gravelly) strata in the grouting process and introducing the power-law slurry viscosity time-varying constitutive equation, the diffusion equation of penetration grouting considering both the tortuosity of the sandy (gravelly) strata and the time-varying slurry viscosity is established.
(2) The diffusion range of power-law fluid in sandy (gravelly) strata shows an obvious nonlinear change with the increase in time. Under three working conditions, the error is the largest without considering the time-varying viscosity of the slurry and the tortuosity effect of sandy (gravelly) strata, which is 71~86% higher than the measured value. Only considering the time-varying viscosity of the slurry and only considering the tortuosity of sandy (gravelly) strata second, which are 53~68% and 23~32% higher than the measured values, respectively. When considering the two factors at the same time, the error is only 13~19%, which verifies the rationality of considering the tortuosity effect of sandy (gravelly) strata and the time-varying viscosity of the power-law slurry.
(3) When the power-law slurry permeates and diffuses in sandy (gravelly) strata, its diffusion range is controlled by the tortuosity of the sandy (gravelly) strata, the water–cement ratio of slurry, and grouting pressure. The tortuosity of the sandy (gravelly) strata is inversely proportional to the diffusion radius of the slurry, and the water–cement ratio of the slurry and grouting pressure are directly proportional to the diffusion radius. The smaller the water–cement ratio of the slurry, the greater the tortuosity of the sandy (gravelly) strata, the greater the resistance encountered by penetration grouting, and the more difficult the diffusion of the slurry.
(4) The main control factors of slurry pressure attenuation in different working conditions are different. For the sandy (gravelly) strata with a small particle size, the tortuosity effect of the sandy (gravelly) strata dominates the slurry pressure attenuation. However, for sandy (gravelly) strata with a large particle size, the main control factor of the slurry pressure attenuation is the tortuosity effect of the sandy (gravelly) strata in the initial stage and the time-varying viscosity of the slurry in the later stage.

## Figures and Tables

**Table 1:** Calculation parameters of the porous media.

Parameter Name	Working Condition	G	γ	ω	$\rho_{p}$	ϕ	$\Gamma \left(\phi \right)$	ϕ′	$\Gamma \left(\phi \prime \right)$
Group	M1	1–3 mm	2.63	3.24	1.63	0.3993	1.3764	0.2747	1.5298
	M2	3–5 mm	2.65	2.79	1.5	0.4505	1.3269	0.2946	1.5011
	M3	5–10 mm	2.72	2.18	1.37	0.5074	1.2781	0.3103	1.4798

Notes: G is the grading of porous media particles; γ is the specific gravity of porous media (g/cm^3^); $\rho_{p}$ is the density of the porous media (g/cm^3^).

**Table 2:** Calculation parameters of the power-law slurry.

Parameter Name	Working Condition	W/C	$\rho_{s}\;(g/{\mathrm{cm}}^{3})$	$n_{0}$	$C_{0}$	λ
Group	M1	0.7	1.67	0.4537	1.8656	0.0009
	M2	0.6	1.75	0.2692	4.4195	0.0010
	M3	0.5	1.84	0.1406	10.4507	0.0011

**Table 3:** Comparison between theoretical value and the measured value of the diffusion radius under different parameters (unit: mm).

Working Condition	Theory L1	Theory L2	Theory L3	Theory L4	Measured Value [20]
M1	127.49	114.06	95.15	85.14	74.2
M2	140.62	129.40	98.46	90.63	79.8
M3	157.22	141.48	111.49	100.34	84.6

## Data Availability

Not Applicable.
